# Healthcare students’ knowledge and attitude toward medical cannabis: a cross-sectional study in southeastern Iran

**DOI:** 10.1186/s42238-026-00424-w

**Published:** 2026-05-11

**Authors:** Mahlagha Dehghan, Samaneh Behzadi Fard, Asma Najmadini, Mansooreh Azizzadeh Forouzi, Morteza Rabiei Vaziri, Amirhossein Shahidizandi

**Affiliations:** 1 HIV/STI Surveillance Research Center, and WHO Collaborating Center for HIV Surveillance, Institute for Futures Studies in Health, Kerman University of Medical Sciences, Kerman, Iran; 2 Reproductive and Family Health Research Center, Kerman University of Medical Sciences, Kerman, Iran; 3 School of Nursing and Midwifery, Lorestan University of Medical Sciences, Lorestan, Iran; 4 Nursing Research Center, Kerman University of Medical Sciences, Kerman, Iran; 5 Neuroscience Research Center, Institute of Neuropharmacology, Kerman University of Medical Sciences, Kerman, Iran

**Keywords:** Medical cannabis, Healthcare, Students, Attitude, Knowledge

## Abstract

**Background:**

Medical cannabis has gained increasing attention globally for its potential in treating various conditions. As the use of medical cannabis expands, it is crucial for future healthcare professionals, particularly physicians, to have a comprehensive understanding of its clinical applications, side effects, and legal status. Therefore, this study aimed to assess the knowledge and attitudes of healthcare students in southeastern Iran regarding medical cannabis.

**Methods:**

In this cross-sectional study (April 4th-June 15th, 2025), 560 healthcare students from Kerman University of Medical Sciences were recruited through convenience sampling (response rate: 90.35%). Data were collected through a demographic and background information questionnaire, the Knowledge about Medical Cannabis-Scale (KMC-S), and the Attitude toward Medical Cannabis-Scale (AMC-S). SPSS 24 facilitated analysis using independent t-tests, ANOVA, Pearson correlation coefficient, and stepwise multivariate regression (α = 0.05).

**Results:**

Participants (66.8% female, mean age = 22.31 ± 2.75 years) demonstrated moderate knowledge (65.6 ± 8.3 vs. midpoint = 60) but cautious attitudes (44.4 ± 5.4 vs. midpoint = 45). While the overall knowledge-attitude correlation was nonsignificant (*r* = -0.008, *p* = 0.855), subscale analysis revealed a weak negative correlation between knowledge and perceptions of “relative advantages to other drugs” (*r* = -0.195, *p* < 0.001) and a positive correlation with perceptions of “Legal and governmental aspects of medical marijuana” (*r* = 0.191, *p* < 0.001). A stepwise multiple regression identified educational year (β = 0.243, *p* < 0.001) and educational training about marijuana (β = 0.179, *p* < 0.001) as key knowledge predictors.

**Conclusion:**

Iranian healthcare students display therapeutic awareness but operational hesitancy regarding cannabis, potentially reflecting cultural-religious reservations. Findings underscore the need for curriculum reforms addressing evidence-based therapeutic applications while considering local sociolegal frameworks. Future research should explore attitude formation mechanisms in conservative medical environments.

**Supplementary Information:**

The online version contains supplementary material available at 10.1186/s42238-026-00424-w.

## Introduction

The term “medical cannabis” (MC) refers to a broad range of cannabinoid-based medicines and preparations used for therapeutic purposes (Pereira et al. 2020). Driven by patient advocacy, regulations governing medical cannabis have evolved significantly worldwide, leading to its growing acceptance in clinical practice (Zolotov et al. 2021). Historically, cannabis has been used for centuries to treat various ailments. The plant contains over 100 different cannabinoids, with Δ9-tetrahydrocannabinol (THC) and cannabidiol (CBD) being the most studied agents responsible for its pharmacological effects (Felnhofer et al. 2021).

Evidence supports the use of cannabis or cannabinoid-based preparations for a variety of medical conditions (Kusturica et al. 2019). For example, they have demonstrated effectiveness in managing chronic pain, alleviating chemotherapy-induced nausea and vomiting, and reducing spasticity in patients with multiple sclerosis (Likhitsathian et al. 2021). However, these therapeutic benefits are balanced by documented adverse effects, including neurocognitive deterioration, memory impairment, and long-term deficits, particularly among children and young adults (Pereira et al. 2020).

Given this complex landscape, the attitudes and knowledge of healthcare students are crucial, as they will be the future health professionals responsible for guiding patients on cannabis-based treatments (Vujcic et al. 2017). However, a significant gap persists in formal medical education, with studies showing medical schools often neglect comprehensive instruction on cannabis therapeutics, leaving students unprepared and prone to relying on non-academic sources (Denneler et al. 2024). Instead of providing a formal curriculum on its medical uses, education is often limited to discussions of its misuse, abuse, and recreational dangers (Pereira et al. 2020; Eiselen et al. 2023; Khamenka and Pikirenia 2021).

Several studies have explored the knowledge and attitudes of healthcare students and physicians towards medical cannabis in various parts of the world. For example, Chan et al. (2016) found that nearly all students (97%) in Colorado believed more research on MC was necessary, acknowledging its potential role in treating various medical conditions (Chan et al. 2017). Similarly, a study by Vujcic et al. (2017) in Serbia highlighted a divide in knowledge, where students who supported legalizing medical cannabis had better knowledge of its medical uses, while opponents were more knowledgeable about its side effects (Vujcic et al. 2017).

While global research on medical cannabis has expanded rapidly, a critical gap remains in understanding how Knowledge and Attitudes manifest in regions with restrictive legal frameworks—particularly the Middle East. Iran’s unique sociopolitical landscape, where cannabis is classified as an illicit substance under national law, creates a dual challenge: future healthcare professionals must reconcile emerging therapeutic evidence with deeply entrenched legal prohibitions. This study conducts the first comprehensive Knowledge and Attitudes assessment among healthcare students in southeastern Iran, a region disproportionately affected by substance use disorders yet underserved in medical cannabis education.

## Materials and methods

### Study design and setting

This cross-sectional study aimed to assess the knowledge and attitude toward medical cannabis in southeastern Iran. The research setting was all medical schools affiliated with Kerman University of Medical Sciences in southeastern Iran.

### Sampling and sample size

The sample size was calculated using Cochran’s formula for proportion estimation (Z_1−α/2_ = 1.96*, p* = 0.5, d = 0.04). According to this formula, a total of 519 people were calculated, and considering the 10% dropout, 560 people were finally calculated.

Convenience sampling was conducted based on permission from institutions and the availability of participants in the selected academic settings. Eligibility criteria included: (1) enrollment in an undergraduate program (Zolotov et al. 2021), and non-visiting student status. Students who declined to participate or submitted incomplete responses were excluded. Questionnaires with more than 10% missing responses or internally inconsistent data were excluded.

A total of 560 self-administered questionnaires were available. After excluding questionnaires with substantial missing data, inconsistent responses, or failure to meet eligibility criteria, 506 questionnaires were retained for final analysis. Therefore, the valid response rate was 90.35%.

### Data collection instruments

Data collection was carried out between April 4th and June 15th, 2025. The data were collected using three instruments: a demographic and background information questionnaire, the Knowledge about Medical Cannabis- Scale (KMC-S), and the Attitude toward Medical Cannabis- Scale (AMC-S).

#### Demographic and background information questionnaire

This questionnaire collected demographic and background information, including age, gender, marital status, living place, major, academic year, family history of drug abuse, friend/classmate history of marijuana use, individual history of Cannabis use, and educational training about Cannabis.

#### Knowledge about Medical Cannabis- Scale (KMC-S)

The KMC-Scale is a 20-item tool developed and validated by the present study researchers to measure healthcare students’ knowledge about medical cannabis (detailed in a separate methodological study currently under peer review). The researchers developed the scale based on a literature review. The response range of the scale includes strongly disagree = 1, disagree = 2, no idea = 3, agree = 4, and strongly agree = 5. There are no inverse items in the scale. The minimum score of the scale is 20, and the maximum score is 100. The higher the score, the more knowledge about medical cannabis.

Ten experts, including one PhD in psychology, two psychiatrists, and seven nursing faculty members, reviewed the scale. Some minor changes were made to most of the items based on the experts’ feedback. The same ten experts determined the CVR and CVI. Based on the CVR, all items had a coefficient above 0.56. Items had a CVI higher than 0.8, with the CVI for the entire scale being 0.901. To check the reliability in the present study, Cronbach’s alpha coefficient and Omega coefficient were calculated. The scale demonstrated good reliability, with a Cronbach’s alpha coefficient of 0.893 and an Omega coefficient of 0.891.

#### Attitude toward Medical Cannabis-Scale (AMC-S)

The AMC-Scale is a 15-item tool developed and validated by the researchers to measure healthcare students’ attitude toward medical cannabis in four key areas: ‘relative advantages to other drugs’ (4 items), ‘general use of Cannabis’ (3 items), ‘legal and governmental aspects of medical Cannabis’ (5 items), and ‘educational aspects of medical Cannabis’ (3 items). The response range of the scale includes strongly disagree = 1, disagree = 2, no idea = 3, agree = 4, and strongly agree = 5. There are no inverse items in the scale. The minimum score of the scale is 15, and the maximum score is 75. The higher the score, the more positive attitude toward medical cannabis.

Content validity ratio and content validity index of the scale were acceptable. The CVI for the entire scale was 0.981. The subscales explained 60.41% of the concept’s variance. The CFA sufficiently confirmed the structure extracted from EFA. Convergent and discriminant validities of the CFA model were confirmed. The scale demonstrated good reliability, with a Cronbach’s alpha coefficient of 0.820 and an Omega coefficient of 0.811. Like the previous scale, the researchers developed the scale based on a literature review, detailed in a separate methodological study currently under peer review (Appendix file 1).

### Data collection

All participants were informed of the study objectives, their rights to voluntary participation, confidentiality of data, and that their academic or professional status would not be affected. Data collection was carried out between April 4th and June 15th, 2025. Paper questionnaires were distributed in person at the participating institutions, and completion took approximately 10–15 min. All distributed questionnaires were checked for completeness and accuracy before data analysis entry.

### Data analysis

SPSS 24 was utilized for data analysis. Descriptive statistics, including frequency, percentage, mean, and standard deviation, were employed to describe the background characteristics of the samples and the main variables of the study. As the data had a normal distribution, an independent t-test and analysis of variance (ANOVA) were employed to investigate the differences in knowledge and attitude scores according to the qualitative variables. In addition, the Pearson correlation coefficient was used to assess the association between knowledge and attitude scores. Stepwise multivariate regression analysis was conducted to further explore the predictive variables of knowledge and attitude scores. In different regression models, knowledge and attitude scores were included as the dependent variable, and all variables with *p* < 0.25 in the bivariate analysis were included as independent variables. In addition, the Mahalanobis d^2^ index was examined to check the multivariate outliers. Accordingly, for knowledge score there was no outliers, therefore, the regression analysis conducted on 506 samples. For attitude score, 17 outliers were excluded from the analysis (Mahalanobis d^2^ index > 13.82), therefore, the regression analysis conducted on 489 samples. The univariate normality of the main variables was checked using the skewness and kurtosis indices. Multicollinearity was confirmed using VIF (Variance Inflation Factor) < 10 (ranged between 1.011 and 1.044) and Tolerance > 0.1 (ranged between 0.967 and 0.988). The Durbin-Watson test was also within an acceptable range (1.714 for knowledge and 2.06 for attitude), which indicates there was no autocorrelation detected in the sample. A significance level of < 0.05 was considered.

## Results

The majority of the participants were female, single, and held a B.Sc. degree. The mean age was 22.31 ± 2.75 (range: 18–44). The major of 31.6% of the participants was medicine. 9.5% of the participants had a positive family history of drug abuse, 12.1% had a positive friend/classmate history of using Cannabis, and 5.1% had a positive individual history of using Cannabis (Table 1).

**Table 1:** The participants’ characteristics and correlation with knowledge and attitude toward medical cannabis (*n* = 506)

Variable	N (%)	Knowledge about medical cannabis		knowledge and attitude toward medical cannabis	
		**Mean (SD)**	**Statistical test (*P* value)**	**Mean (SD)**	**Statistical test (*P* value)**
Age (yr.)					
18–20	135 (26.7)	63.19 (7.83)	*F* = 5.762 (0.001)	44.24 (4.95)	*F* = 3.649 (0.013)
21–23	239 (47.3)	66.28 (7.97)		43.88 (5.64)	
24–26	107 (21.1)	67.20 (9.11)		45.95 (5.71)	
> 26	25 (4.9)	65.92 (8.58)		44.52 (4.80)	
Gender					
Female	338 (66.8)	65.37 (8.19)	t = −0.994 (0.321)	43.96 (5.05)	t = −2.852 (0.005)
Male	168 (33.2)	66.15 (8.61)		45.43 (6.18)	
Marital Status					
Single	454 (89.6)	65.65 (8.40)	*F* = 1.213 (0.298)	44.53 (5.45)	*F* = 1.286 (0.277)
Married	47 (9.3)	66.09 (7.79)		44.04 (5.94)	
Divorced/Widowed	5 (1.0)	60.00 (6.40)		40.80 (1.80)	
Living place					
Kerman	305 (60.3)	65.25 (8.48)	t = −1.286 (0.199)	44.17 (5.69)	t = −1.389 (0.165)
Others	201 (39.7)	66.22 (8.10)		44.87 (5.15)	
Major					
Medicine	160 (31.6)	67.10 (8.89)	*F* = 5.137 (< 0.001)	44.76 (5.53)	*F* = 3.043 (0.006)
Nursing	86 (17.0)	63.17 (7.26)		44.37 (5.58)	
Pharmacology	66 (13.0)	69.08 (6.99)		43.51 (5.45)	
Dentistry	45 (8.9)	64.09 (7.59)		43.36 (5.00)	
Midwifery	34 (6.7)	63.91 (7.68)		42.85 (4.38)	
Anesthesiology	27 (5.3)	65.81 (9.53)		47.85 (5.64)	
Others	88 (17.5)	64.18 (8.29)		44.80 (5.53)	
Educational year					
1	141 (27.9)	63.61 (7.60)	*F* = 7.591 (< 0.001)	44.06 (5.16)	*F* = 1.003 (0.423)
2	65 (12.8)	63.48 (7.50)		44.78 (5.49)	
3	106 (20.9)	64.81 (7.78)		45.19 (5.94)	
4	93 (18.4)	66.80 (8.35)		43.97 (5.13)	
5	62 (12.3)	69.66 (9.25)		44.14 (5.90)	
6	24 (4.7)	67.62 (7.56)		43.96 (5.54)	
7	15 (3.0)	72.73 (9.07)		46.47 (5.32)	
Family history of drug abuse					
Yes	48 (9.5)	68.14 (8.90)	t = 2.205 (0.028)	46.33 (5.46)	t = 2.514 (0.012)
No	458 (90.5)	65.37 (8.24)		44.25 (5.46)	
Friend/classmate’s history of Cannabis use					
Yes	61 (12.1)	67.64 (9.71)	t = 2.012 (0.045)	45.90 (5.43)	t = 2.214 (0.029)
No	445 (87.9)	65.36 (8.10)		44.25 (5.47)	
Individual history of Cannabis use					
Yes	26 (5.1)	68.54 (8.84)	t = 1.830 (0.068)	48.00 (4.84)	t = 3.424 (0.001)
No	480 (94.9)	65.48 (8.29)		44.26 (5.46)	
Educational training about Cannabis					
Yes	49 (9.7)	71.00 (8.14)	t = 4.848 (< 0.001)	42.86 (6.14)	t = −2.144 (0.033)
No	457 (90.3)	65.06 (8.16)		44.62 (5.39)	

*SD* Standard deviation, *F* Analysis of Variance, *t* Independent t test

Healthcare students’ mean score for knowledge was 65.63 ± 8.33, which is above the scale’s midpoint of 60. The two highest-scoring knowledge items were that Cannabis users can become addicted to it (4.18 ± 0.92) and Cannabis can impair a person’s ability to drive (4.15 ± 0.089). The two lowest-scoring knowledge items were: “I have sufficient information to answer patients’ questions about the safety of Cannabis” (2.10 ± 1.03) and “I have sufficient information to answer patients’ questions about the effectiveness of Cannabis” (2.12 ± 1.08) (Table 2).

**Table 2:** Knowledge about medical cannabis among healthcare students (*n* = 506)

Item	Mean	SD
1. I have sufficient information about the appropriate use of Cannabis for medical purposes	2.21	1.15
2. I have sufficient information to answer patients’ questions about the effectiveness of Cannabis	2.12	1.08
3. I have sufficient information to answer patients’ questions about the safety of Cannabis	2.10	1.03
4. There are no clinical guidelines for the use of Cannabis for medical purposes	2.55	0.99
5. The risks and benefits of potential therapeutic uses of Cannabis are not sufficiently clear	3.10	0.10
6. I do not have sufficient knowledge, education, and personal information about the use of Cannabis for medical purposes	2.98	0.93
7. There are possible side effects while using Cannabis	3.97	0.88
8. There is a possible interaction of Cannabis use with other medications	3.96	0.91
9. I understand the difference between tetrahydrocannabinol (THC) and cannabidiol (CBD)	2.44	1.17
10. I am not sure whether Cannabis has medicinal value	3.11	0.97
11. Cannabis users can become addicted to it	4.18	0.92
12. Cannabis can impair a person’s ability to drive	4.15	0.89
13. Cannabis use can lead to dry mouth	3.62	0.84
14. Cannabis use can lead to dry eyes	3.55	0.80
15. Cannabis use can lead to an increased risk of oral cannabis-induced stomatitis (inflammation of the mouth tissues and white growths in the oral cavity)	3.48	0.75
16. Inhalation of Cannabis can lead to an increased risk of lung/esophageal tissue irritation	3.61	0.77
17. Inhalation of Cannabis can reduce pain perception	3.65	0.78
18. Inhalation of Cannabis can lead to an increased risk of cancer	3.57	0.76
19. Cannabis use can lead to increased levels of anxiety	3.62	0.81
20. Inhalation of Cannabis can lead to a decrease in short-term cognitive function	3.68	0.83
Total	65.63	8.33

The mean score of attitudes toward MC was 44.45 ± 5.49 among healthcare students, which was lower than the midpoint of the scale, i.e., 45. The highest attitude score belonged to “Education regarding the medical use of Cannabis is necessary,” with 4.03 ± 0.88, and “Physicians must receive formal training on the medical use of Cannabis before recommending it to patients,” with 4.02 ± 0.89, respectively. The lowest attitude score belonged to “Recreational Cannabis use is safe,” with 2.07 ± 1.04, and “Cannabis should be legalized for the general public,” with 2.16 ± 1.00, respectively (Table 3).

**Table 3:** Attitude toward medical cannabis among healthcare students (*n* = 506)

Item	Mean	SD
1. Cannabis has fewer negative health effects compared to opioid medications	2.77	0.75
2. Cannabis has fewer negative health effects compared to tobacco	2.76	0.77
3. Cannabis has fewer negative health effects compared to alcohol	2.80	0.78
4. Cannabis has fewer negative health effects compared to other prescription drugs	2.68	0.77
Relative advantages to other drugs	11.01	2.30
5. Cannabis should be legalized for the general public	2.16	1.00
6. Recreational Cannabis use is safe	2.07	1.04
7. All physicians should be authorized to prescribe medical Cannabis	2.49	0.96
General use of Cannabis	6.72	2.49
8. Cannabis should be legalized for medicinal purposes	3.08	0.73
9. With support from the Ministry of Health, medical Cannabis prescriptions can be issued more easily	3.13	0.82
10. Pharmacists must participate in the distribution process of medical Cannabis	3.20	0.99
11. If I had to decide today about legalizing medical Cannabis use, I would support it	2.63	0.98
12. Our government has sufficient resources to regulate the medical use of Cannabis	2.68	0.93
Legal and governmental aspects of medical Cannabis	14.73	2.84
13. Education regarding the medical use of Cannabis is necessary	4.03	0.88
14. Physicians must receive formal training on the medical use of marijuana before recommending it to patients	4.02	0.89
15. Physicians should maintain continuous communication with patients to whom they recommend Cannabis	3.94	0.88
Educational aspects of medical Cannabis	11.99	2.28
Total score	44.45	5.49

There was no significant correlation between knowledge and attitude toward MC among healthcare students (*P* = 0.855). However, there was a negative weakly significant correlation between knowledge and the subscale of “Relative advantages to other drugs” and a positive weakly significant correlation between knowledge and the subscale of “Legal and governmental aspects of medical Cannabis” (*P* < 0.001) (Table 4).

**Table 4:** Correlation between knowledge and attitude toward medical cannabis among healthcare students (*n* = 506)

Variable		Knowledge about Medical Cannabis	
		**r**	***P* value**
Attitude toward Medical Cannabis	Relative advantages to other drugs	−0.049	0.273
	Relative advantages to other drugs	−0.195	< 0.001
	General use of Cannabis	0.042	0.350
	Legal and governmental aspects of medical Cannabis	0.191	< 0.001
	Total	−0.008	0.855

In addition, bivariate analysis showed that the knowledge score about MC had a significant association with age, education major, educational year, family history of drug abuse, friend/classmate history of using Cannabis, and educational training about Cannabis (Table 1). Also, the attitude score toward MC had a significant association with age, gender, education major, family history of drug abuse, friend/classmate history of using Cannabis, individual history of Cannabis use, and educational training about Cannabis (Table 1).

A stepwise multiple regression was employed to explore predictors of knowledge score about MC among the participants. Age, living place, education major, educational year, family history of drug abuse, friend/classmate history of using Cannabis, individual history of Cannabis use, and educational training about Cannabis were included in the model as independent variables. The model explained 9.9% of the variance in knowledge score about MC was explained by educational year and educational training about Cannabis. Specifically, for every one-year increase in educational years, the knowledge score about MC increased by 1.208 points. Those who attended educational training about Cannabis scored 5.048 points higher in the knowledge score about the MC score than those who did not (Table 5).

**Table 5:** Regression coefficients of the effect of underlying variables on knowledge and attitude toward medical cannabis

Dependent variable	Independent variable	b	S.E	β	t	*P* value	Confidence interval of 95%
Knowledge about Medical Cannabis^*^	Educational year	1.208	0.211	0.243	5.716	< 0.001	0.793–1.623
	Educational training about Cannabis (No = 0, Yes = 1)	5.048	1.199	0.179	4.209	< 0.001	2.692–7.404
Attitude toward Medical Cannabis^**^	Individual history of Cannabis use (No = 0, Yes = 1)	3.195	1.106	0.130	2.888	0.004	1.021–5.369
	Educational major (Anesthesiology vs. Medicine)	3.308	1.070	0.137	3.092	0.002	1.206–5.411
	Gender (Female = 0, Male = 1)	1.254	0.526	0.107	2.386	0.017	0.221–2.287
	Educational training about Cannabis (No = 0, Yes = 1)	−1.885	0.814	−0.102	−2.316	0.021	−3.484 – −0.286

^*^Adjusted *R*^2^ = 0.099, *F* = 28.829 (*P* < 0.001)

^**^Adjusted *R*^2^ = 0.057, *F* = 8.411 (*P* < 0.001)

A stepwise multiple regression was employed to explore predictors of attitude score toward MC among the participants. Age, gender, living place, education major, family history of drug abuse, friend/classmate history of using Cannabis, individual history of Cannabis use, and educational training about Cannabis were included in the model as independent variables. The model explained 5.7% of the variance in attitude score about MC was explained by individual history of Cannabis use, education major, gender, and educational training about Cannabis. Specifically, those who had a positive individual history of Cannabis use scored 3.195 points higher in attitude score about MC than those who had not. Those who were studying anesthesiology scored 3.308 points higher in attitude score about MC than those who were studying medicine. Males scored 1.254 points higher in attitude score about MC than females. Those who attended educational training about Cannabis scored 1.885 points lower in attitude score about MC than those who did not (Table 5).

## Discussion

This study aimed to assess the knowledge and attitudes of healthcare students toward medical cannabis in southeastern Iran. Our results indicated that healthcare students had a foundational but non-uniform knowledge of medical cannabis. Knowledge was strongest concerning the well-documented negative effects of cannabis, such as addiction and impaired driving, which aligns with findings from Dolatshahi et al. (2023). However, students scored lowest on items related to the clinical application of this knowledge, which is consistent with similar studies. For example, Orjuela‑Rojas et al. (2021) found that a significant majority of Colombian psychiatrists were unsure how to legally help patients access medical cannabis (Orjuela-Rojas et al. 2021). Similarly, studies by Piava et al. (2024) and Denneler et al. (2024) highlighted a lack of awareness about the endocannabinoid system and a perception among healthcare students that cannabis is dangerous and unsuitable for medical use (Paiva et al. 2024; Denneler et al. 2024). Additionally, in a systematic review, Weisman & Rodríguez (2021) found that healthcare students and professionals consistently emphasized a strong need for expanded medical cannabis education, while also voicing significant worries about its potential for dependency and addiction (Weisman and Rodríguez 2021).

The overall attitude score was just below the scale’s midpoint, suggesting a prevailing sense of caution and skepticism. This is particularly evident in students’ strong support for formal education on medical cannabis for physicians, coupled with a negative view of recreational use and broad public legalization. This result is consistent with a study by Jacobs et al. (2022) and Khamenka et al. (2021), both of whom reported a lack of formal medical cannabis training among healthcare students (Jacobs et al. 2022; Khamenka and Pikirenia 2021). Our results are also supported by Kaikoushi et al. (2022), who found that a majority of healthcare students opposed the legalization of cannabis for recreational use (Kaikoushi et al. 2022). In contrast, a study by Findley et al. (2021) in the US and Israel revealed that secular students were significantly more likely to support recreational legalization than their religious counterparts (Findley et al. 2021), indicating that cultural and religious factors may influence attitudes. In interpreting how religion influences students’ attitudes toward medical cannabis, it can be said that many faith systems explicitly classify psychoactive substances as haram (forbidden) or morally corrupting.

A key finding of our study is the lack of a significant correlation between a student’s knowledge and their attitude toward medical cannabis. This suggests that simply increasing a student’s knowledge about the substance does not necessarily lead to a more positive or permissive attitude. This result aligns with a study by Felnhofer et al. (2021), which showed that healthcare students were skeptical about whether physicians should prescribe and offer it to patients (Felnhofer et al. 2021). Religious-legal norms create a knowledge-attitude gap, while outdated curricula fail to address ethical dilemmas or legal risks. Students recognize global evidence but face institutional bans and skill gaps, forming a triple barrier to medical cannabis integration. However, this contrasts with a study by Likhitsathian et al. (2021), which found that Israeli healthcare students had a more permissive attitude toward MC but were less prepared to answer patients’ questions about MC compared to their Thai peers (Likhitsathian et al. 2021).

Our regression analysis provided valuable insight into the predictors of knowledge and attitude. We found that academic year and specific educational training about Cannabis were the strongest predictors of knowledge, confirming that formal learning experiences are crucial for building a knowledge base. While training increased knowledge, it was associated with a lower attitude score. This aligns with findings from Chung et al. (2022), which showed that final-year students recognized a significantly higher number of indications and side effects than third-year students (Chung et al. 2022). However, this contrasts with a study from Serbia by Kusturica et al. (2019), which reported that clinical teaching and hospital training did not influence students’ knowledge of medical cannabis (Kusturica et al. 2019). Interestingly, while training increased knowledge, it was associated with a lower attitude score, suggesting that a deeper understanding of the complexities and potential risks of medical cannabis may lead to a more cautious stance.

### Limitations

This study provides novel insights into medical cannabis knowledge and attitudes among Iranian healthcare students, yet several key limitations warrant consideration. The indigenous scales (AMC-S) and (KMC-S), while demonstrating acceptable internal consistency, await peer-reviewed validation of their psychometric properties. Convenience sampling from Kerman Medical University limits generalizability to broader medical populations. The cross-sectional design precludes analysis of how evolving cannabis legislation might influence attitude trajectories, while self-report methods risk residual social desirability biases despite anonymous data collection. To address these constraints, future research should implement longitudinal designs tracking knowledge evolution pre-post policy reforms, triangulate self-reports with prescription pattern analyses.

## Conclusion

This study highlights an important gap in medical education: while students understand basic facts about medical cannabis, many feel unprepared to discuss it with patients. Our findings show that simply teaching the science isn’t enough—future healthcare professionals need hands-on training to build real-world skills. We recommend three practical changes: 1) Add workshops where students practice answering tough questions about cannabis risks and benefits, 2) Create partnerships with pain clinics so students can observe cannabis treatment plans in action, and 3) Develop clear guidelines for managing common concerns like addiction or cultural stigma. These steps would help turn textbook knowledge into confident patient care, especially as Iran updates its medical cannabis policies.

## Supplementary Information

Supplementary Material 1.

## Data Availability

The data are available upon request to the corresponding author after signing the appropriate documents in line with ethical application and the decision of the Ethics Committee.

## Abbreviations

MC: Medical Cannabis
THC: Δ9-Tetrahydrocannabinol
CBD: Cannabidiol
CVI: Content Validity Index
CFA: Confirmatory Factor Analysis
EFA: Exploratory Factor Analysis
VIF: Variance Inflation Factor
ANOVA: Analysis of Variance

## References

[CR1] Chan MH, Knoepke CE, Cole ML, McKinnon J, Matlock DD. Colorado medical students’ attitudes and beliefs about marijuana. J Gen Intern Med. 2017;32(4):458–63. 28097606 10.1007/s11606-016-3957-y PMC5377890

[CR2] Chung AKK, Tse CY, Law JKC. Attitudes and beliefs of medical students on cannabis in Hong Kong. Complement Ther Med. 2022;70:102870. 35952958 10.1016/j.ctim.2022.102870

[CR3] Denneler T, Mahling M, Hermann S, Stengel A, Zipfel S, Herrmann-Werner A, et al. Medical students’ attitudes and perceived competence regarding medical cannabis and its suggestibility. BMC Med Educ. 2024;24(1):149. 38360743 10.1186/s12909-024-05089-8 PMC10867999

[CR4] Dolatshahi S, Nezhadi A, Jahangiri J, Dolatshahi B. Attitudes toward marijuana use among public university students in Tehran. Int J High Risk Behav Addict. 2023;12(4):e139679.

[CR5] Eiselen E, Naidu K, Viljoen M. Attitudes of medical students regarding legalisation of cannabis and cannabis-education. S Afr J Psychiatr. 2023;29:1948. 38059193 10.4102/sajpsychiatry.v29i0.1948 PMC10696579

[CR6] Felnhofer A, Kothgassner OD, Stoll A, Klier C. Knowledge about and attitudes towards medical cannabis among Austrian university students. Complement Ther Med. 2021;58:102700. 33677020 10.1016/j.ctim.2021.102700

[CR7] Findley PA, Edelstein OE, Pruginin I, Reznik A, Milano N, Isralowitz R. Attitudes and beliefs about medical cannabis among social work students: cross-national comparison. Complement Ther Med. 2021;58:102716. 33812039 10.1016/j.ctim.2021.102716

[CR8] Jacobs RJ, Colon J, Kane MN. Medical students’ attitudes, knowledge, and beliefs about medical cannabis: a qualitative descriptive study. Cureus. 2022;14(8):e28336. 10.7759/cureus.28336 PMC9502535 36168342

[CR9] Kaikoushi K, Alexandrou G, Rousou E, NM MK. Attitudes, beliefs and knowledge regarding medical cannabis among healthcare students in Cyprus: a cross-sectional descriptive correlational study. 2022. 10.3389/fpsyt.2023.1196915 PMC10375702 37520218

[CR10] Khamenka N, Pikirenia U. Knowledge, attitudes and beliefs about medical cannabis among the medical students of the Belarus State Medical University. Complement Ther Med. 2021;57:102670. 33486047 10.1016/j.ctim.2021.102670

[CR11] Kusturica MP, Tomas A, Sabo A, Tomić Z, Horvat O. Medical cannabis: knowledge and attitudes of prospective doctors in Serbia. Saudi Pharm J. 2019;27(3):320–5. 30976174 10.1016/j.jsps.2018.11.014 PMC6438677

[CR12] Likhitsathian S, Edelstein OE, Srisurapanont M, Zolotov Y, Karawekpanyawong N, Reznik A, et al. Cross national comparison of medical students’ attitudes and beliefs about medical cannabis and its application for pain management. Complement Ther Med. 2021;59:102720. 33864906 10.1016/j.ctim.2021.102720

[CR13] Orjuela-Rojas JM, GarcíaOrjuela X, Ocampo Serna S. Medicinal cannabis: knowledge, beliefs, and attitudes of Colombian psychiatrists. J Cannabis Res. 2021;3(1):26. 34225825 10.1186/s42238-021-00083-z PMC8259442

[CR14] Paiva C, Santos T, Cunha-Oliveira A, Rosendo I, Pita J-R. Knowledge, opinions and experiences of nurses and nursing students in Portugal regarding the use of medical cannabis. BMC Nurs. 2024;23(1):788. 39468501 10.1186/s12912-024-02443-5 PMC11520810

[CR15] Pereira L, Núñez-Iglesias MJ, Domínguez-Martís EM, López-Ares D, González-Peteiro M, Novío S. Nursing students’ knowledge and attitudes regarding medical marijuana: a descriptive cross-sectional study. Int J Environ Res Public Health. 2020;17(7):2492. 32268474 10.3390/ijerph17072492 PMC7177422

[CR16] Vujcic I, Pavlovic A, Dubljanin E, Maksimovic J, Sipetic-Grujicic S. Attitudes toward medical cannabis legalization among Serbian medical students. Subst Use Misuse. 2017;52(9):1229–35. 10.1080/10826084.2017.1302959 28605305

[CR17] Weisman JM, Rodríguez M. A systematic review of medical students’ and professionals’ attitudes and knowledge regarding medical cannabis. J Cannabis Res. 2021;3(1):47. 34641976 10.1186/s42238-021-00100-1 PMC8507207

[CR18] Zolotov Y, Metri S, Calabria E, Kogan M. Medical cannabis education among healthcare trainees: a scoping review. Complement Ther Med. 2021;58:102675. 33539943 10.1016/j.ctim.2021.102675

