# Supplementary material for: Healthcare students’ knowledge and attitude toward medical cannabis: a cross-sectional study in southeastern Iran
Source: J Cannabis Res. 2026 May 11;8:80. doi: 10.1186/s42238-026-00424-w (PMC13334929; doi:10.1186/s42238-026-00424-w)
Supplement: Supplementary file 1 — Supplementary Material 1. [file 42238_2026_424_MOESM1_ESM.docx]

**Appenddix File 1**

**Exploratory Factor Analysis**

Table 1. Rotated factor matrix of the Attitude toward Medical Cannabis scale

| **No** | **Items** | **Factor Load** | | | | **Communalities** | |
| --- | --- | --- | --- | --- | --- | --- | --- |
|  |  | **Factor 1** | **Factor 2** | **Factor 3** | **Factor 4** | **Initial** | **Extraction** |
| 2 | Education regarding the medical use of marijuana is necessary. |  |  |  | 0.664 | 0.459 | 0.457 |
| 3 | Physicians must receive formal training on the medical use of marijuana before recommending it to patients. |  |  |  | 0.949 | 0.651 | 0.913 |
| 4 | Physicians should maintain continuous communication with patients to whom they recommend marijuana. |  |  |  | 0.722 | 0.555 | 0.581 |
| 5 | Marijuana should be legalized for medicinal purposes. |  |  | 0.430 |  | 0.372 | 0.358 |
| 9 | With more education, I can better treat patients using medical marijuana. |  |  | 0.534 |  | 0.344 | 0.356 |
| 11 | With support from the Ministry of Health, medical marijuana prescriptions can be issued more easily. |  |  | 0.670 |  | 0.456 | 0.508 |
| 13 | Pharmacists must participate in the distribution process of medical marijuana. |  |  | 0.642 |  | 0.475 | 0.455 |
| 16 | I would support the legalization of medical marijuana if I had to make that decision today. |  |  | 0.630 |  | 0.518 | 0.578 |
| 17 | The government has adequate resources for regulating medical marijuana. |  |  | 0.447 |  | 0.374 | 0.359 |
| 6 | The medical use of marijuana is safe. |  | 0.498 |  |  | 0.311 | 0.309 |
| 7 | Marijuana should be legalized for the general public. |  | 0.809 |  |  | 0.568 | 0.666 |
| 8 | Recreational marijuana use is safe. |  | 0.787 |  |  | 0.581 | 0.652 |
| 12 | All physicians should be authorized to prescribe medical marijuana. |  | 0.552 |  |  | 0.378 | 0.408 |
| 15 | Medical marijuana use during pregnancy is safe. |  | 0.459 |  |  | 0.386 | 0.371 |
| 18 | Marijuana has fewer negative health effects compared to opioid medications. | 0.569 |  |  |  | 0.531 | 0.532 |
| 19 | Marijuana has fewer negative effects than tobacco. | 0.809 |  |  |  | 0.649 | 0.743 |
| 20 | Marijuana has fewer adverse effects than alcohol. | 0.766 |  |  |  | 0.559 | 0.628 |
| 21 | Marijuana has fewer negative effects compared to other drugs. | 0.661 |  |  |  | 0.476 | 0.503 |
| 22 | The use of marijuana has significant benefits for physical health. | 0.418 |  |  |  | 0.406 | 0.397 |
|  | **Eigenvalue** | 5.940 | 2.732 | 1.546 | 1.260 |  |  |
|  | **Explained variance** | 31.266 | 14.377 | 8.139 | 6.629 |  |  |
|  | **Cumulative variance** | 60.411 | | | |  |  |

**Comfirmatory Factor Analysis and Convergent and Discriminant Validity**

The PCMIN/DF ratio (χ²/df = 1.556), RMSEA = 0.051, CFI = 0.965, GFI = 0.926, IFI = 0.966, and NFI = 0.910 were within the acceptable range. Only AGFI was slightly less than the acceptable level (0.893). Therefore, the CFA model can confirm the structure identified in the exploratory factor analysis. Therefore, the final scale consists of 15 items with four factors: the first factor with 4 items (#items 18, 19, 20, and 21), the second factor with 3 items (#items 7, 8, and 12), the third factor with five items (#items 5, 11, 13, 16, and 17), and the fourth factor with 3 items (#items 2, 3, 4) .

Furturmore, we checked the convergent and discriminant validity of the factors in CFA model. All AVE values were ≥ 0.50, and CR values were ≥ 70, confirming convergent validity. Both ASV and MSV values were lower than AVE (Table 4). In addition, the HTMT Ratio was calculated for all factors. All HTMT ratios ragned from 0.001 (F1🡪F4) to 0.641 (F2🡪 F3). These results support the disciminant validity of the model .

Table 2. Findings of convergent and discriminant validity

|  | Total score | | AVE | CR | MSV | ASV |
| --- | --- | --- | --- | --- | --- | --- |
|  | R | P-value |  |  |  |  |
| Factor 1 | 0.697 | <0.001 | 0.789 | 0.868 | 0.403 | 0.236 |
| Factor 2 | 0.630 | <0.001 | 0.531 | 0.785 | 0.489 | 0.267 |
| Factor 3 | 0.837 | <0.001 | 0.625 | 0.809 | 0.403 | 0.346 |
| Factor 4 | 0.351 | <0.001 | 0.763 | 0.766 | 0.382 | 0.052 |

R: Spearman’s rank correlation coefficient; AVE: average variance extracted; CR: composite reliability; MSV: Maximum Shared Variance; ASV: Average Shared Variance

**Reliability**

The reliability of the 15-item scale was assessed using Cronbach’s alpha and McDonald’s omega coefficients in a sample of 300 participants. The Cronbach’s alpha was 0.820 for the overall scale and above 0.720 for all subscales. The McDonald’s omega coefficient was 0.811 for the entire scale and above 0.0.777 for all subscales.

**Table 3.** The Cronbach’s alpha and McDonald’s omega coefficients of the Attitude toward Medical Cannabis scale

| **Subscales** | **Cronbach’s alpha correlation coefficient** | **McDonald’s omega correlation coefficient** |  |
| --- | --- | --- | --- |
| **Factor 1 (4 items)** | 0.843 | 0.848 |  |
| **Factor 2 (3 items)** | 0.720 | 0.795 |  |
| **Factor 3 (5 items)** | 0.769 | 0.777 |  |
| **Factor 4 (3 items)** | 0.827 | 0.839 |  |
| **Total Scale** | 0.820 | 0.811 |  |
